# Yes-Associated Protein 1 as a Novel Prognostic Biomarker for Gastrointestinal Cancer: A Meta-Analysis

**DOI:** 10.1155/2018/4039173

**Published:** 2018-11-14

**Authors:** Luo Zhang, Xing Song, Xiaodong Li, Changping Wu, Jingting Jiang

**Affiliations:** ^1^Department of Tumor Biological Treatment, The Third Affiliated Hospital of Soochow University, Changzhou, Jiangsu, China; ^2^Department of Oncology, The Third Affiliated Hospital of Soochow University, Changzhou, Jiangsu, China

## Abstract

**Background:**

Yes-associated protein 1 (YAP1) is an effector of Hippo pathway, which plays a significant role in cell proliferation and tumor progression. The relationship between YAP1 and gastrointestinal cancer has been explored in many previous studies. We conducted a meta-analysis to explore the prognostic effect of YAP1 in patients with gastrointestinal cancer.

**Methods:**

A systematic search was performed through the PubMed, Web of Science, Embase, and Cochrane library databases to collect eligible studies. The pooled hazard ratios (HRs) with 95% confidence intervals (CIs) were used to evaluate the relationship between YAP1 expression and gastrointestinal cancer clinical outcomes.

**Results:**

A total of 2941 patients from 18 studies were enrolled. The results showed that elevated YAP1 expression predicted a poor prognosis in gastrointestinal cancer (HR = 1.56; 95% CI: 1.29-1.89;* P* < 0.001). Subgroup analyses indicated significant association between YAP1 overexpression and shorter OS of patients with esophageal squamous cell carcinoma (HR = 1.85; 95% CI: 1.25-2.73;* P* = 0.002), gastric cancer (HR = 1.41,95% CI: 1.02-1.95;* P* = 0.037), and colorectal cancer (pooled HR = 1.75; 95% CI: 1.42-2.15;* P* < 0.001). However, YAP1 expression did not affect DFS of patients with gastrointestinal cancer (pooled HR = 1.33; 95% CI: 0.95-1.88;* P* = 0.101).

**Conclusion:**

Elevated YAP1 expression in patients with gastrointestinal cancer might be related to shorter OS. YAP1 protein could serve as a potential predictor of poor prognosis in gastrointestinal cancer.

## 1. Introduction

Gastrointestinal cancer is one of the major malignant diseases detrimental to health. Esophageal cancer (EC), gastric cancer (GC), and colorectal cancer (CRC) are the major malignancies of gastrointestinal cancer. EC is the sixth leading cause of cancer-related death worldwide, GC is the third and CRC is the fifth [1]. Although the overall survival (OS) of patients with gastrointestinal cancer has improved due to growing early detection and more widespread implementation of radical surgery, plenty of patients with gastrointestinal cancer continue to be diagnosed at advanced stages and therefore lose the optimal opportunity for radical cure. Therefore, searching for ideal diagnostic and prognostic biomarker is significant to improve the curative effect of gastrointestinal cancer.

Recently, researches on signal transduction pathways have revealed the Hippo-YAP pathway plays a vital part in modulating tissue homeostasis, organ size, and tumor progression. Yes-associated protein 1 (YAP or YAP1), an oncoprotein encoded by the* YAP1* gene located on the human chromosome 11q22, is a transcriptional effector component of the Hippo pathway to regulate cell proliferation and apoptosis [2]. YAP1 was first discovered as an intracellular binding protein and a transcriptional coactivator by Sudol in 1994 [3]. Increasing expression of YAP1 protein was detected in numerous cancers, including esophageal cancer, gastric cancer, and colorectal cancer.

Elevated YAP1 expression was reported to be associated with worse outcomes in gastrointestinal cancer in most studies, but some studies reported the dual functions of YAP1 to promote and inhibit the progression of human malignancies. Suh [4] revealed that YAP1 had a tumor suppressor function in GC. In human colon carcinoma cell line H116, YAP1 interacts with* p73* and increases* p73* transactivation of apoptotic genes [5]. In order to research the relationship between YAP1 protein and prognosis of gastrointestinal cancer, we performed a systematic review and meta-analysis to assess the prognostic implications of elevated YAP1 protein in patients with gastrointestinal cancer.

## 2. Materials and Methods

### 2.1. Literature Search

PubMed, Web of Science, Embase, and Cochrane library databases were searched to retrieve feasible literatures about the theme up to Jul 9, 2018. The key terms used in the search strategy were (“gastrointestinal” or “esophageal” or “gastric” or “stomach” or “colorectal” or “colon”) and (“Yes associated protein 1” or “YAP1” or “Yes associated protein” or “YAP”). The eligibility articles were limited to clinical trials. We went over the full text of identified articles. We searched manually to ensure all available studies were included in this meta-analysis. Two investigators (L. Zhang and X. Song) conducted literature collection independently. Different opinions from two investigators were resolved by consultation.

### 2.2. Study Selection Criteria

Literatures eligible for this meta-analysis were identified according to the following criteria: (1) all patients were diagnosed as EC or GC or CRC by pathological examination; (2) YAP1 protein expression in human tumor tissues was detected; (3) the prognostic value of YAP1 in patients with gastrointestinal cancer was investigated; (4) the hazard ratio (HR) and corresponding 95% confidence interval (CI) could be extracted for overall Survival (OS) or disease-free survival (DFS). The exclusion criteria for articles included (1) non-English language articles; (2) case reports, letters, reviews, conference abstracts, and animal experiments; (3) insufficient data available to estimate outcomes; (4) size of each study arm less than 10 participants; (5) selecting the latest or complete study if one patient cohort was researched in more than one studies.

### 2.3. Quality Assessment and Data Collection

The Newcastle-Ottawa Quality Assessment Scale (NOS) was used to assess the quality of the studies and a score ≥ 6 was regarded as high quality. Two researchers extracted requisite information from all the eligible studies independently, including first author's surname, year of publication, nationality, case number, detected method, outcome measure, follow-up months, the cut-off value, HR, and corresponding 95% CI. We selected multivariate result if multivariate and univariate results were both reported in a study.

### 2.4. Statistical Analysis

All meta-analyses were performed with STATA 12.0 Software (Stata Corporation, College Station, TX, USA), and* P* < 0.05 indicated statistical significance unless otherwise specified. The main outcome was OS or DFS. Pooled HRs with 95% CIs were used to quantitatively determine the association between positive YAP1 expression and clinical prognosis. If the study had reported HR and 95% CI, we extracted them directly. Otherwise, they were determined by the data extracted from Kaplan-Meier survival curves using Engauge Digitizer version 4.1 [6]. We also contacted to the corresponding authors of relevant articles if needed. A pooled HR greater than 1 indicated a shorter survival for the patients with positive YAP1 expression, while HR less than 1 suggested a longer survival. The statistical heterogeneity was measured by the I^2^ statistic and Chi-square test (*P* value) [7, 8]. If* P* ≤ 0.05 or* I*^*2*^ ≥ 50%, indicating a problem with statistically heterogeneity, we chose the random-effects model to conduct analyses. In contrast, we used the fixed-effects model. Meta-regression and subgroup analyses were performed to further explore the potential source of heterogeneity. Funnel plot, Egger's test, and Begg's test were performed to evaluate the publication bias [9].

## 3. Results

### 3.1. Study Characteristics

**Figure 1:**
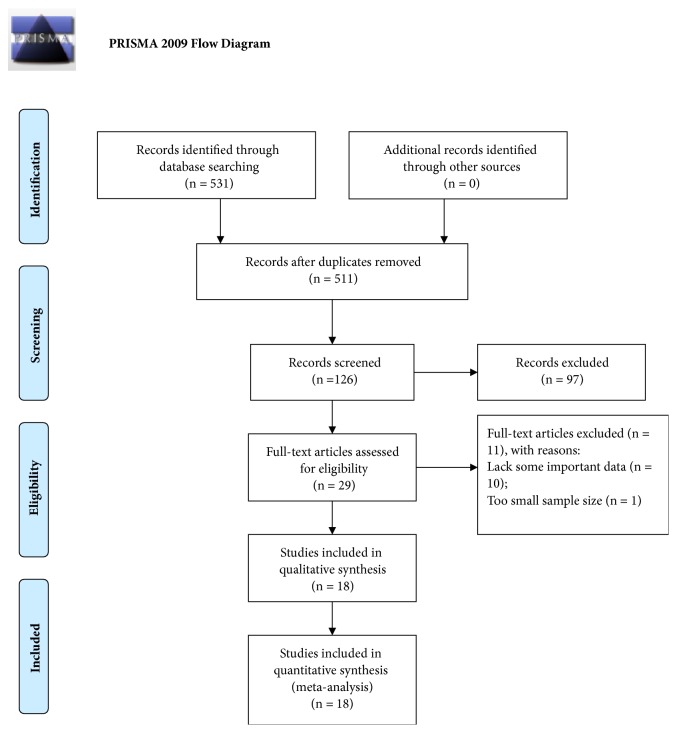
Flow diagram of the studies selection process.

Our search strategy primarily retrieved 531 records. 385 papers were directly excluded by reading the titles and abstracts. After looking through full text of every paper, 97 articles were excluded because they were not related to YAP1 protein or did not study human gastrointestinal cancer. In the remaining 29 papers, 11 papers were excluded for the following reasons: lack some important data (n = 10) or too small sample size (n = 1). Finally, 18 articles published from 2012 to 2018 studied the effect of positive YAP1 expression on patients survival in gastrointestinal cancer were enrolled into this meta-analysis (Figure 1) [4, 10–26]. The main information of the enrolled studies is showed in Table 1. The 18 studies included 2 studies detecting YAP1 expression in esophageal squamous cell carcinoma (ESCC), 9 in GC, and 7 in CRC. A total of 2941 patients diagnosed with gastrointestinal cancer were included. 11 studies (62.50%) reported on Chinese, 7 studies (31.25%) on Koreans, and only 1 study (6.25%) on Japanese. The YAP1 protein was detected by Immunohistochemistry (IHC) in all the 18 studies, but these studies reported different cut-off values. HRs and 95% CIs for OS and/or DFS were provided directly in 12 studies and estimated from Kaplan-Meier Survival curves in the other 6 studies. According to the NOS, all included articles were of high quality (score≥7), with a mean of 7.67. Thus, all studies were eligible for this meta-analysis.

### 3.2. Overall Survival

**Figure 2:**
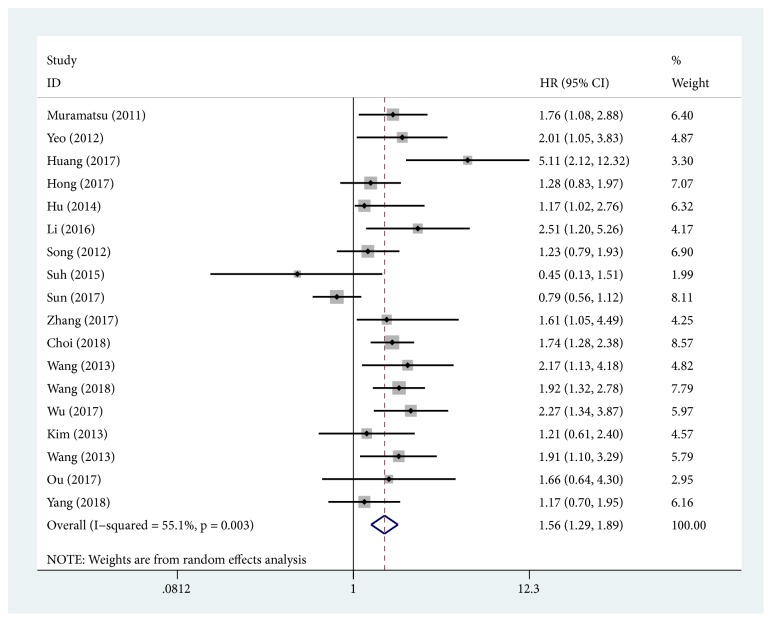
Forest plots of studies evaluating hazard ratios of high YAP1 expression in gastrointestinal cancer for overall survival.

**Figure 3:**
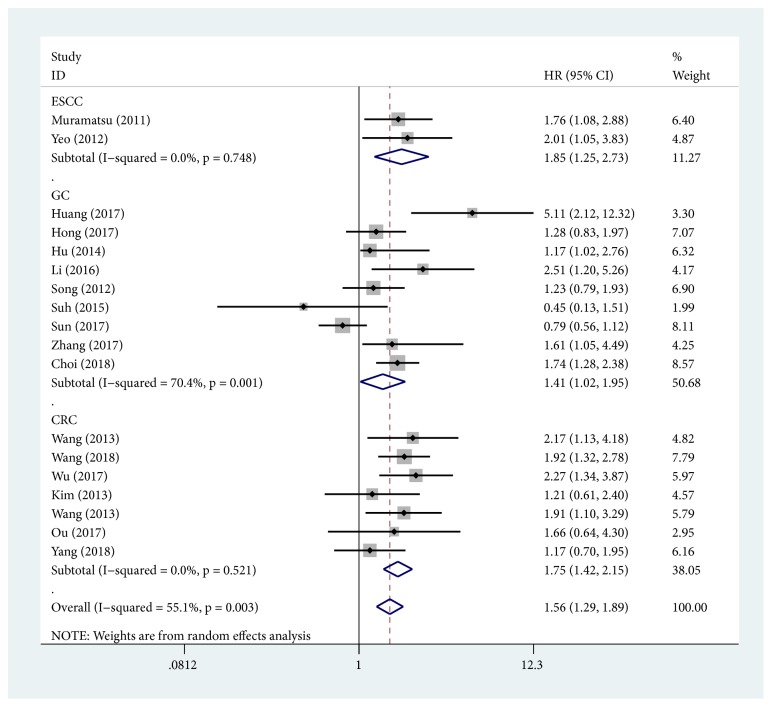
Forest plot of the relationship between high YAP1 expression and overall survival in patients with a variety of cancers.

18 studies provided sufficient information for OS or DFS analysis. The key results of this meta-analysis are shown in Table 2. Because of the heterogeneity between studies in evaluating OS (*I*^*2*^ = 55.1%,* P* = 0.003), a random-effects model was used to pool the HRs. The pooled analysis indicated that higher YAP1 expression was significantly associated with shorter OS in gastrointestinal cancer patients (HR = 1.56; 95% CI: 1.29-1.89;* P* < 0.001) (Figure 2). The tumor type subgroup analysis demonstrated negative impact of elevated YAP1 on OS in patients with ESCC (HR = 1.85; 95% CI: 1.25-2.73;* P* = 0.002), GC (HR = 1.41,95% CI: 1.02-1.95;* P* = 0.037), and CRC (pooled HR = 1.75; 95% CI: 1.42-2.15;* P* < 0.001) (Figure 3). In regard to country subgroup analysis, higher expression of YAP1 was visibly associated with shorter OS in Japanese patients (pooled HR = 1.76; 95% CI: 1.08-2.88;* P* = 0.024), Chinese patients (pooled HR =1.70; 95% CI: 1.26-2.29;* P* < 0.001), and Korean patients (pooled HR = 1.41; 95% CI: 1.10-1.80;* P* = 0.007). For OS, pooled HR values > 1 were still calculated in subgroup meta-analyses stratified by case number, HR obtained method, and analysis type (Table 2).

### 3.3. Sensitivity Analyses and Publication Bias

**Figure 4:**
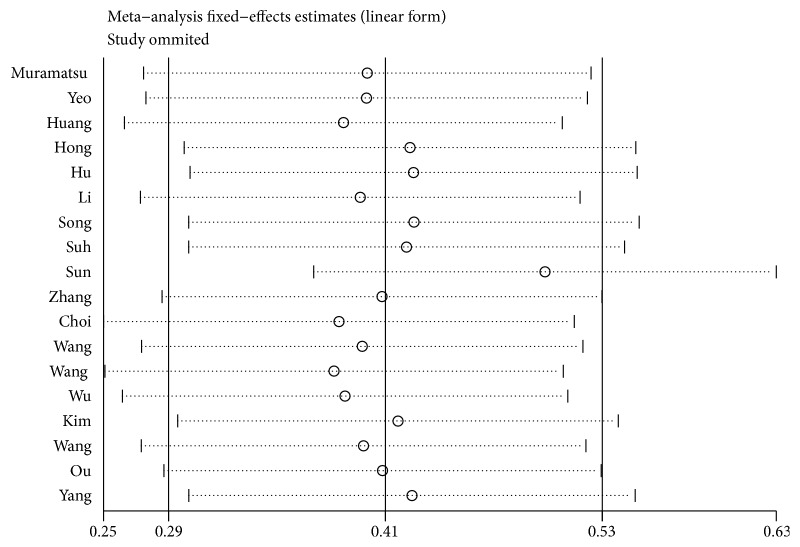
Sensitivity analysis on the relationships between YAP1 expression and overall survival in gastrointestinal cancer patients.

**Figure 5:**
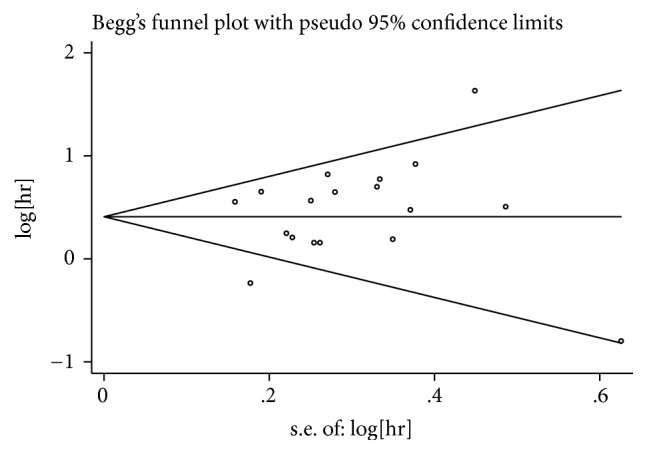
Funnel plots of publication biases on the relationships between YAP1 expression and overall survival in gastrointestinal cancer patients.

We used the random-effects model to assess sensitivity analysis by sequential omission of individual studies, and the outcome was unaffected by any single study (Figure 4). Then we performed a meta-regression to detect the potential causes for the heterogeneity. The results showed no statistically significant impact of country (*P* = 0.639), tumor type (*P* = 0.779), sample size (*P* = 0.405), analysis type (*P* = 0.830), HR obtained method (*P* = 0.830), or cut-off value (*P* = 0.326) on the combined effect size for OS. Funnel plots, Egger's test, and Begg's test were used to assess the publication bias of all enrolled studies. Funnel plot (Figure 5) suggested no publication bias existed, and Egger's test supported the same result (*P* = 0.37). Thus, the results of this meta-analysis were dependable.

### 3.4. Disease-Free Survival

**Figure 6:**
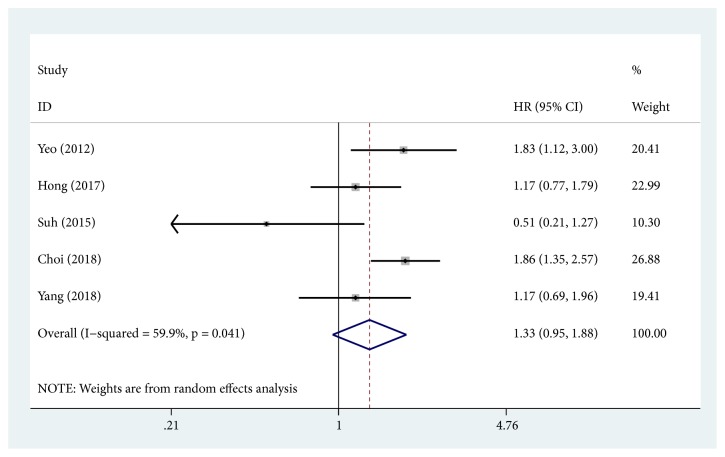
Forest plots of studies evaluating hazard ratios of high YAP1 expression in gastrointestinal cancer for disease-free survival.

5 studies enrolled a total of 869 patients from China and Korea, providing suitable information for DFS analyses. Because of obvious statistical heterogeneity (*I*^2^ = 59.9%;* P* = 0.041) (Table 3), a random-effects model was used to calculate the pooled HR. The result revealed no association between higher level of YAP1 and shorter DFS (pooled HR = 1.33; 95% CI = 0.95–1.88;* P* = 0.101). A forest plot of study-specific HRs for DFS is presented in Figure 6. All the HRs and corresponding 95% CIs are shown in Table 3. Subgroup analysis of cancer type revealed the adverse effect of elevated YAP1 on DFS in patients with ESCC (pooled HR = 1.83; 95% CI = 1.12-3.00;* P* < 0.001), but there was no correlation between YAP1 expression and DFS in GC or CRC patients. Moreover, the association between YAP1 and DFS was not significant in both Chinese (pooled HR= 1.17, 95% CI = 0.69-1.97) and Korean (pooled HR = 1.35, 95% CI = 0.89-2.06) populations.

## 4. Discussion

The Hippo signaling pathway was first identified and named by screening for mutant tumor suppressors in flies, and it was revealed that tumor progressed due to increased cell proliferation and decreased cell apoptosis when the components of the Hippo pathway had loss-of-function mutations [27]. The Hippo pathway is strongly participated in several processes of cancer progression. Evidence shows that the Hippo pathway is interconnected with other cancer-relevant pathways, especially the transforming growth factor-*β* (TGF-*β*), G protein-coupled receptors (GPCRs), and WNT pathways [28].

YAP1 and PDZ-binding motif (TAZ) are the downstream proteins in the Hippo pathway. These two proteins are in charge of controlling cell proliferation and have important regulatory effects on stem cell self-renewal, tissue regeneration, and organ development. YAP1 contains 488 amino acids and several structural domains [29]. The most crucial domains are a TEA DNA-binding domain and two-WW domain. The former binds to the TEA Domain Transcription factor (TEAD), while the latter binds to a transcriptional coactivator, which in turn binds to the PPxY motif present on transcription factors [30]. TAZ, the YAP1 homologous protein, is identified as a 14-3-3 binding protein. It has similarity structure and biological functions [31]. Normally, phosphorylated YAP/TAZ is accumulated in the cytoplasm, maintaining a highly conservative state and without transcriptional kinase activity. Under pathological conditions, the Hippo pathway which is blocked or inactivated loses of the effect on phosphorylating YAP/TAZ. Then YAP/TAZ is combined with the transcription factor TEAD. The complex transfers to the nucleus and promotes the transcription of corresponding genes, resulting in the imbalance between cell proliferation and apoptosis; eventually these changes lead to cell over proliferation and even cause the tumor occurring [32, 33]. Although YAP1 is usually identified as an oncoprotein with functions of enhancing the tumor cells survival, migration, tumor angiogenesis, and chemotherapy resistance, some literature supports the idea that YAP1 functions as a tumor suppressor in cancers, for instance, head and neck cancers (HNC) [34], breast cancer [35], hematological malignancy [36], and CRC [5].

Gastrointestinal cancer is one of the major health care problems in the world. We performed the first meta-analysis to provide strong evidence to reveal the prognostic implications of YAP1 in gastrointestinal cancer. Elevated YAP1 protein expression was obviously correlated with shorter OS in patients with gastrointestinal cancer. In the subsequent subgroup analyses, the adverse prognostic role of elevated YAP1 expression remained stable in different country, sample size, HR obtained method, and analysis type. Nevertheless, the existing evidence from included studies was insufficient to prove a definitive correlation between YAP1 and DFS. Our findings showed YAP1 was not an independent prognostic factor for DFS in gastrointestinal cancer. Nonetheless, because of the significant heterogeneity, the results should be treated with caution. This heterogeneity might be partly due to the variation in patients' selection among the studies. For instance, the proportion of advanced stage patients was different from study to study. Stratified analyses according to clinicopathological characteristics (such as anatomic site and disease stage) were not performed in this meta-analysis for the limitation of available original studies. The analyses can be conducted in the future to further assess the relationship between YAP1 and DFS in gastrointestinal cancer with more available studies.

Furthermore, previous research shows elevated YAP1 expression is also related with shorter relapse free survival (RFS) in gastrointestinal cancer [12, 21]. In addition to YAP1 protein, elevated expression of* YAP1* mRNA may also predict worse prognosis in patients with gastrointestinal cancer [37]. YAP1 also plays a role in early diagnosis of gastrointestinal cancer. Da [38] reported that detecting YAP1 and surviving together might help in early diagnosis of gastric carcinoma.

However, there are some deficiencies for this meta-analysis as well. First, there was a problem of heterogeneity in the overall analysis and in some subgroup analyses. Subgroup analyses revealed that the heterogeneity might be due to the different characteristics of the tumor types. Second, the different cut-off values of YAP1 IHC detection might impact on the precision of the prognostic role of YAP1 in gastrointestinal cancer. In order to establish the most suitable cut-off value, further well-designed studies with larger sample size are imperative to carry out. Third, it might be insufficient to provide support for all ethnic groups because all the studies included were carried out in Asian population.

## 5. Conclusions

In conclusion, our results suggest that YAP1 protein overexpression correlates with shorter OS in gastrointestinal cancer, especially in Asian patients. Furthermore, YAP1 has been considered to be a promising target for therapy of gastrointestinal cancer. Considering of the deficiencies of the present paper, further well-designed, prospective, and national multicenter, large sample researches are imperative to verify the clinical value of YAP1 in gastrointestinal cancer.

## Figures and Tables

**Table 1:** Main characteristics of all studies included in the meta-analysis.

**First author **	**Year**	**Country**	**tumor type**	**Case number**	**Follow-up (months)**	**Detected method**	**Cut-off value**	**Multivariate analysis**	**HRs provided from**	**Outcome measures**	**NOS score**
Muramatsu	2011	Japan	ESCC	120	1-103	IHC	≥30% of cells stained	yes	Report	OS	7
Yeo	2012	Korea	ESCC	142	over60	IHC	IRS ≥4	yes	Report	OS/DFS	7
Huang	2017	China	GC	120	longest96	IHC	IRS ≥4	yes	Report	OS	9
Hong	2017	Korea	GC	166	0-119	IHC	≥10% of cells stained	no	SC	OS/DFS	8
Hu	2014	China	GC	214	longest65	IHC	IRS ≥4	yes	Report	OS	8
Li	2016	China	GC	161	median34	IHC	IRS >2	yes	Report	OS	8
Song	2012	Korea	GC	223	5-75	IHC	≥50% of cells stained	yes	Report	OS	7
Suh	2015	Korea	GC	116	3-51	IHC	≥10% of cells stained	no	SC	OS/DFS	7
Sun	2017	China	GC	270	107	IHC	≥10% of cells stained	yes	Report	OS	9
Zhang	2017	China	GC	178	80	IHC	IRS ≥3	no	SC	OS	8
Choi	2018	Korea	GC	300	over60	IHC	IRS ≥6	no	SC	OS/DFS	8
Wang	2013	China	CRC	168	1-56	IHC	IRS ≥4	yes	Report	OS	8
Wang	2018	China	CRC	172	longest50	IHC	IRS ≥4	no	SC	OS	7
Wu	2017	China	CRC	85	102 to 2572 days	IHC	H-score >150	yes	Report	OS	7
Kim	2013	Korea	CRC	132	longest125	IHC	IRS ≥7	yes	Report	OS	7
Wang	2013	China	CRC	139	over60	IHC	≥10% of cells stained	yes	Report	OS	8
Ou	2017	China	CRC	90	longest60	IHC	IRS ≥2	no	SC	OS	7
Yang	2018	China	CRC	145	longest125	IHC	IRS ≥2	yes	Report	OS/DFS	8

ESCC: esophageal squamous cell carcinoma; GC: gastric cancer; CRC: colorectal cancer; IHC: immunohistochemistry; IRS: immunoreactivity score; HR: hazard ratio; SC: survival curve; OS: overall survival; DFS: disease free survival; NOS:Newcastle-Ottawa Scale.

**Table 2:** Pooled HR for OS of patients with high expression of YAP1 according to subgroup analyses.

Outcome subgroup	No. of patients	No. of studies	Fixed-effects model		Heterogeneity	
			HR (95% CI)	*P* value	*I* ^2^ (%)	*P*
OS	2941	18	1.56(1.29,1.89)	<0.001	55.1	0.003
Tumor type						
ESCC	262	2	1.85(1.25,2.73)	0.002	0	0.748
GC	1748	9	1.41(1.02,1.95)	0.037	70	0.001
CRC	931	7	1.92(1.32,2.78)	<0.001	0	0.521
Contry						
China	1742	11	1.70(1.26,2.29)	<0.001	67.1	0.001
Korea	1079	6	1.41(1.10,1.80)	0.007	27.7	0.227
Japan	120	1	1.76(1.08,2.88)	0.024	-	-
Case number						
<150	1089	9	1.72(1.27,2.33)	<0.001	47	0.057
≥150	1852	9	1.45 (1.13,1.86)	0.003	60	0.009
HR obtained method						
Reported	2085	12	1.61(1.24,2.10)	<0.001	64.2	0.001
SC	856	6	1.58(1.25,2.00)	<0.001	21.1	0.275
Analysis type						
Multivariate	2085	12	1.61(1.24,2.10)	<0.001	64.2	0.001
Univariate	856	6	1.58(1.25,2.00)	<0.001	21.1	0.275

OS: overall survival; ESCC: esophageal squamous cell carcinoma; GC: gastric cancer; CRC: colorectal cancer; HR: hazard ratio; CI: confidence interval; SC: survival curve.

**Table 3:** Pooled HR for DFS of patients with high expression of YAP1 according to subgroup analyses.

Outcome subgroup	No. of patients	No. of studies	Fixed-effects model		Heterogeneity	
			HR (95% CI)	*P* value	*I* ^2^ (%)	*P*
DFS	869	5	1.33(0.95,1.88)	<0.001	59.9	0.041
Tumor type						
ESCC	142	1	1.83(1.12,3.00)	0.016	-	-
GC	582	3	1.18(0.66,2.11)	0.584	76.4	0.014
CRC	145	1	1.17(0.69,1.88)	0.556	-	-
Contry						
China	145	1	1.35(0.89,2.06)	0.556	-	-
Korea	724	4	1.17(0.95,1.88)	0.159	67.4	0.027

DFS: disease-free survival; ESCC: esophageal squamous cell carcinoma; GC: gastric cancer; CRC: colorectal cancer; HR: hazard ratio; CI: confidence interval.

## References

[1] Torre L. A., Bray F., Siegel R. L., Ferlay J., Lortet-Tieulent J. (2015). Global cancer statistics, 2012. *CA: A Cancer Journal for Clinicians*.

[2] Rosenbluh J., Nijhawan D., Cox A. (2012). *β*-Catenin-Driven Cancers Require a YAP1 Transcriptional Complex for Survival and Tumorigenesis. *Cell*.

[3] Sudol M. (1994). Yes-Associated Protein (YAP65) is a proline-rich phosphoprotein that binds to the SH3 domain of the Yes proto-oncogene product. *Oncogene*.

[4] Suh J. H., Won K. Y., Kim G. Y. (2015). Expression of tumoral FOXP3 in gastric adenocarcinoma is associated with favorable clinicopathological variables and related with Hippo pathway. *International Journal of Clinical and Experimental Pathology*.

[5] Levy D., Adamovich Y., Reuven N., Shaul Y. (2007). The Yes-associated protein 1 stabilizes p73 by preventing Itch-mediated ubiquitination of p73. *Cell Death & Differentiation*.

[6] Wang Y., Zeng T. (2013). Response to: Practical methods for incorporating summary time-to-event data into meta-analysis. *Trials*.

[7] Higgins J. P. T., Thompson S. G. (2002). Quantifying heterogeneity in a meta-analysis. *Statistics in Medicine*.

[8] Higgins J. P. T., Thompson S. G., Deeks J. J., Altman D. G. (2003). Measuring inconsistency in meta-analyses. *British Medical Journal*.

[9] Zhang L., Song X., Shao Y., Wu C., Jiang J. (2018). Prognostic value of Midkine expression in patients with solid tumors: a systematic review and meta-analysis. *Oncotarget *.

[10] Muramatsu T., Imoto I., Matsui T. (2011). YAP is a candidate oncogene for esophageal squamous cell carcinoma. *Carcinogenesis*.

[11] Yeo M.-K., Kim S.-H., Kim J. M. (2012). Correlation of expression of phosphorylated and non-phosphorylated yes-associated protein with clinicopathological parameters in esophageal squamous cell carcinoma in a korean population. *Anticancer Reseach*.

[12] Huang S., Zhu L., Cao Y. (2017). Significant association of YAP1 and HSPC111 proteins with poor prognosis in Chinese gastric cancer patients. *Oncotarget *.

[13] Hong S. A., Son M. W., Cho J. (2017). Low angiomotin-p130 with concomitant high Yes-associated protein 1 expression is associated with adverse prognosis of advanced gastric cancer. *APMIS-Acta Pathologica, Microbiologica et Immunologica Scandinavica*.

[14] Hu X., Xin Y., Xiao Y., Zhao J. (2014). Overexpression of YAP1 is Correlated with Progression, Metastasis and Poor Prognosis in Patients with Gastric Carcinoma. *Pathology & Oncology Research*.

[15] Li P., Sun D., Li X. (2016). Elevated expression of Nodal and YAP1 is associated with poor prognosis of gastric adenocarcinoma. *Journal of Cancer Research and Clinical Oncology*.

[16] Song M., Cheong J.-H., Kim H., Noh S. H., Kim H. (2012). Nuclear expression of Yes-associated protein 1 correlates with poor prognosis in intestinal type gastric cancer. *Anticancer Reseach*.

[17] Sun D., Li X., He Y. (2016). YAP1 enhances cell proliferation, migration, and invasion of gastric cancer in vitro and in vivo. *Oncotarget *.

[18] Zhang B., Gong A., Shi H. (1894). Identification of a novel YAP-14-3-3zeta negative feedback loop in gastric cancer. *Oncotarget *.

[19] Wang L., Shi S., Guo Z. (2013). Overexpression of YAP and TAZ Is an Independent Predictor of Prognosis in Colorectal Cancer and Related to the Proliferation and Metastasis of Colon Cancer Cells. *PLoS ONE*.

[20] Wang Q., Gao X., Yu T. (2018). REGgamma controls Hippo signaling and reciprocal NF-kappaB-YAP regulation to promote colon cancer. *Clinical Cancer Research*.

[21] Wu D.-W., Lin P.-L., Wang L., Huang C.-C., Lee H. (2017). The YAP1/SIX2 axis is required for DDX3-mediated tumor aggressiveness and cetuximab resistance in KRAS-wild-type colorectal cancer. *Theranostics*.

[22] Kim D. H., Kim S. H., Lee O. J. (2013). Differential expression of Yes-associated protein and phosphorylated Yes-associated protein is correlated with expression of Ki-67 and phospho-ERK in colorectal adenocarcinoma. *Histology and Histopathology*.

[23] Wang Y., Xie C., Li Q., Xu K., Wang E. (2013). Clinical and prognostic significance of Yes-associated protein in colorectal cancer. *Tumor Biology*.

[24] Ou C., Sun Z., Li X. (2017). MiR-590-5p, a density-sensitive microRNA, inhibits tumorigenesis by targeting YAP1 in colorectal cancer. *Cancer Letters*.

[25] Choi W., Kim J., Park J. YAP/TAZ initiates gastric tumorigenesis via upregulation of MYC. *Cancer Research*.

[26] Yang R., Cai T., Wu X. (2018). Tumour YAP1 and PTEN expression correlates with tumour-associated myeloid suppressor cell expansion and reduced survival in colorectal cancer. *The Journal of Immunology*.

[27] Pan D. (2010). The hippo signaling pathway in development and cancer. *Developmental Cell*.

[28] Kriz V., Korinek V. (2018). WNT, RSPO and hippo signalling in the intestine and intestinal stem cells. *Gene*.

[29] Zhao B., Li L., Lei Q., Guan K. (2010). The Hippo-YAP pathway in organ size control and tumorigenesis: an updated version. *Genes & Development*.

[30] Moroishi T., Hansen C. G., Guan K.-L. (2015). The emerging roles of YAP and TAZ in cancer. *Nature Reviews Cancer*.

[31] Gandhirajan R. K., Jain M., Walla B. (2016). Cysteine S-glutathionylation promotes stability and activation of the Hippo downstream effector transcriptional Co-activator with PDZ-binding motif (TAZ). *The Journal of Biological Chemistry*.

[32] Saucedo L. J., Edgar B. A. (2007). Filling out the Hippo pathway. *Nature Reviews Molecular Cell Biology*.

[33] Harvey K. F., Zhang X., Thomas D. M. (2013). The Hippo pathway and human cancer. *Nature Reviews Cancer*.

[34] Ehsanian R., Brown M., Lu H. (2010). YAP dysregulation by phosphorylation or Δnp63-mediated gene repression promotes proliferation, survival and migration in head and neck cancer subsets. *Oncogene*.

[35] Yu S. J., Hu J. Y., Kuang X. Y. (2013). MicroRNA-200a promotes anoikis resistance and metastasis by targeting YAP1 in human breast cancer. *Clinical Cancer Research*.

[36] Cottini F., Hideshima T., Xu C. (2014). Rescue of Hippo coactivator YAP1 triggers DNA damage-induced apoptosis in hematological cancers. *Nature Medicine*.

[37] Lee K.-W., Lee S. S., Hwang J.-E. (2016). Development and validation of a six-gene recurrence risk score assay for gastric cancer. *Clinical Cancer Research*.

[38] Da C.-L., Xin Y., Zhao J., Luo X.-D. (2009). Significance and relationship between Yes-associated protein and survivin expression in gastric carcinoma and precancerous lesions. *World Journal of Gastroenterology*.

